# Quantitation of 5-Methyltetrahydrofolic Acid in Dried Blood Spots and Dried Plasma Spots by Stable Isotope Dilution Assays

**DOI:** 10.1371/journal.pone.0143639

**Published:** 2015-11-25

**Authors:** Markus Kopp, Michael Rychlik

**Affiliations:** 1 Chair for Analytical Food Chemistry, Technische Universität München, Freising, Germany; 2 Research Center for Nutrition and Food Science, Technische Universität München, Freising, Germany; CSIR- Indian Institute of Toxicology Research, INDIA

## Abstract

Because of minimal data available on folate analysis in dried matrix spots (DMSs), we combined the advantages of stable isotope dilution assays followed by LC-MS/MS analysis with DMS sampling to develop a reliable method for the quantitation of plasma 5-methyltetrahydrofolic acid in dried blood spots (DBSs) and dried plasma spots (DPSs) as well as for the quantitation of whole blood 5-methyltetrahydrofolic acid in DBSs. We focused on two diagnostically conclusive parameters exhibited by the plasma and whole blood 5-methyltetrahydrofolic acid levels that reflect both temporary and long-term folate status. The method is performed using the [^2^H_4_]-labeled isotopologue of the vitamin as the internal standard, and three steps are required for the extraction procedure. Elution of the punched out matrix spots was performed using stabilization buffer including Triton X-100 in a standardized ultrasonication treatment followed by enzymatic digestion (whole blood only) and solid-phase extraction with SAX cartridges. This method is sensitive enough to quantify 27 nmol/L whole blood 5-methyltetrahydrofolic acid in DBSs and 6.3 and 4.4 nmol/L plasma 5-methyltetrahydrofolic acid in DBSs and DPSs, respectively. The unprecedented accurate quantification of plasma 5-methyltetrahydrofolic acid in DBSs was achieved by thermal treatment prior to ultrasonication, inhibiting plasma conjugase activity. Mass screenings are more feasible and easier to facilitate for this method in terms of sample collection and storage compared with conventional clinical sampling for the assessment of folate status.

## Introduction

Folates, particularly folylpolyglutamates, are coenzymes for methyl-, formyl-, and other single-carbon functional group transfer, and are involved in DNA component, protein, and neurotransmitter synthesis [1]. A key metabolic pathway for folates is the methylation cycle, wherein 5-methyltetrahydrofolate (5-CH_3_-H_4_folate) is one of the main donors of single-carbon groups, particularly in homocysteine to methionine remethylation [1]. These essential functions underline the importance of adequate folate supply. Folate deficiency is associated with neural tube defects in newborns [2] and is thought to be involved in cardiovascular diseases [3] and neurodegenerative diseases such as Alzheimer’s disease [4]; it may also be involved in the development of certain forms of cancer [5]. When assessing folate deficiency, individual genetic diversity needs to be taken into account with inadequate dietary folate intake. The C667T single nucleotide polymorphism of methylenetetrahydrofolate reductase (MTHFR) leads to amino acids Ala→Val substitution at amino acid 222 and results in a thermally unstable and less active enzyme responsible for less 5,10-methylenetetrahydrofolic acid conversion to 5-CH_3_-H_4_folate and an increase in nonmethylated folates, particularly in homozygous individuals [6,7]. Therefore, the 5-CH_3_-H_4_folate quantitation in blood samples is suitable for estimating and characterizing the potential risks resulting from folate undersupply and genetic background. Erythrocytes, which maintain their folate content for their 3-month lifespan, are commonly used as biomarkers for long-term folate supply, whereas plasma reflects short-term folate status [8,9]. The 5-CH_3_-H_4_folate-to-tetrahydrofolate (H_4_folate) ratio in whole blood may be a characteristic parameter in blood samples for MTHFR polymorphism determination. Conversely, plasma samples are the perfect tools for short-term folate bioavailability studies because an increase in plasma folate levels is observed shortly after folate intake. However, folate analysis is quite challenging because folate shows low stability in the presence of oxygen [10] and light [11], and its concentration in blood and plasma samples is low. Stable isotope dilution assays (SIDAs) conducted using LC–MS/MS have proven their superiority over other methods used so far [12–15]. Conversely, dried blood spot (DBS) sampling has gained increasing importance in connection with LC–MS since its development in 1963 by Guthrie and Suzi for newborn screenings [16]. Along with these neonatal screenings, metabolic profiling and drug monitoring are other methods based on dried blood or plasma spots [17]. Therefore, compared with conventional methods, sensitive SIDAs based on LC–MS/MS are good tools when combined with the small sample amounts in dried matrix spots (DMS) owing to their low limits of detection (LODs) and quantification (LOQs). The DMS sampling technique offers an opportunity for cost reduction as no medical surveillance is necessary for this less invasive blood sampling method, which could even be performed at home [18]. Number of animals for preclinical studies could also be reduced; easier storage and shipping are feasible with DBS technology compared with conventional methods [19]. Another advantage of blood spotting is preservation of the natural state of blood. Erythrocytes and enzymes remain stable after drying at room temperature, whereas blood sample freezing leads to analyte and blood constituent degradation [20].

Because there are few reports on the folate determination in human blood and plasma spots [20–22], we aimed to develop a new innovative method. All the analytical methods used are based on folate analysis in erythrocytes and plasma by SIDAs using LC–MS/MS as previously described [9]. Our first priority was to reduce the necessary sample amount and establish a sensitive method for the quantitation of 5-CH_3_-H_4_folate in DBSs for whole blood folate status assessment. Thereafter, we focused on the quantitation of 5-CH_3_-H_4_folate in DPSs and DBSs for plasma folate status assessment. The envisaged method development was successful and concluded by several applications as proofs-of-principle.

## Materials and Methods

### Ethics Statement

Blood sampling of volunteers and the preliminary study on folate bioavailability were performed after written informed consent of the volunteers with permission of the Ethics Commission of the TUM School of Medicine of the Technische Universität München (project 4031/11).

### Chemicals

Rat serum and chicken pancreas containing γ-glutamyl hydrolase (EC 3.4.19.9) were obtained from Biozol (Eching, Germany) and Difco (Sparks, MD, USA), respectively. Acetonitrile, potassium dihydrogen phosphate, disodium hydrogen phosphate (anhydrous), methanol, sodium chloride, sodium acetate trihydrate, and sodium hydroxide were purchased from Merck (Darmstadt, Germany). Dithiothreitol (DTT) was purchased from Applichem (Darmstadt, Germany). Formic acid, 4-morpholineethanesulfonic acid (MES) and Triton X-100 were obtained from Sigma (Deisenhofen, Germany). Ascorbic acid was purchased from VWR Chemicals Prolabo (Leuven, Belgium). The isotopological internal standards [^2^H_4_]-5-CH_3_-H_4_folate for 5-CH_3_-H_4_folate quantification and [^2^H_4_]-H_4_folate for H_4_folate quantification were synthesized as previously reported [23] and revealed a content of the respective unlabeled analyte below 0.5%. 5-CH_3_-H_4_folate was purchased from Schircks Laboratories (Jona, Switzerland). One-way micro lancets (Accu Check Safe-T-Pro Plus) were obtained from Roche Diagnostics (Mannheim, Germany). Whatman 903 Protein Saver Cards were purchased from GE Healthcare (Westborough, MA, USA), and Microvette tubes for blood sampling were purchased from Sarstedt (Nümbrecht, Germany). Micro glass capillaries (50 μl) were obtained from Hirschmann (Eberstadt, Germany), and Strata SAX cartridges (quaternary amine, 100 mg, 1 ml) were obtained from Phenomenex (Aschaffenburg, Germany). Folate 400 was obtained from Pure Encapsulations (Sudbury, MA, USA).

### Solutions

Extraction buffer 1 for DBS comprised 0.1% Triton X-100 in a 20 g/L ascorbic acid and MES (200 mmol/L) solution with 0.1 g DTT and was adjusted to pH 5 with 7.5 M NaOH. Extraction buffer 2 for DPS comprised a 20 g/L ascorbic acid and 200 mmol/L MES solution with 0.1 g DTT and was adjusted to pH 5 with 7.5 M NaOH. Phosphate buffer (100 mmol/L) was prepared by adjusting an 100 mmol/L disodium hydrogen phosphate aqueous solution with an potassium dihydrogen phosphate (100 mmol/L) aqueous solution to pH 7.0. The equilibration buffer for the SAX cartridges was prepared by adding 0.02 g DTT to diluted phosphate buffer (10 mmol/L). Further, the eluting solution was a mixture of aqueous sodium chloride (5%) and aqueous sodium acetate (100 mmol/L) containing 0.1 g DTT and ascorbic acid (1%). The chicken pancreas suspension for folylpolyglutamate deconjugation was prepared by stirring chicken pancreas (10 mg) in aqueous phosphate buffer solution (60 ml, 100 mmol/L) containing 1% ascorbic acid adjusted to pH 7 with 7.5 M NaOH.

Rat serum was aliquoted and stored at -20°C without further dilution.

### Sampling and Spotting Procedure

Blood sampling was performed by the forefinger punction using a one-way micro lancet. The first blood drop was discarded, and finger blood was collected in EDTA-Microvette tubes to avoid clotting. Aliquots of blood (50 μl) were spotted on the imprinted DBS circles with 50 μl heparinized end-to-end capillaries and then dried for 2.5 h in the dark. Venous plasma (vP) was collected by conventional venous blood sampling with subsequent blood cell and plasma separation. Aliquots (30 μl) were spotted on the imprinted circles of the Protein Saver Card, which had been coated with 1% aqueous ascorbic acid as previously described [22]. The cards were immersed in 1% ascorbic acid solution for 30 s and then dried for 2 h. The spots were dried for 3 h in the dark. The dried blood and plasma spots were stored at –20°C with desiccant in resealable plastic bags until analysis.

### Dried Blood Spots

DBSs (50 μl) were punched out entirely with a 3-mm hand puncher and spiked with 0.78 ng of the deuterated internal standard [^2^H_4_]-5-CH_3_-H_4_folate. Subsequently, the mixture was suspended in 1 ml of extraction buffer 1 and placed in an ultrasonic bath with ice for 60 min in the dark. Afterward, the sample was mixed with 75 μl rat serum and 1 ml chicken pancreas suspension for enzymatic deconjugation. After constant agitation in a water bath at 37°C for 4 h, the samples were heated in boiling water for 4 min, cooled in ice, and then centrifuged at 4,000 rpm (20 min, 4°C). The supernatant was then subjected to solid-phase extraction (SPE) as described below.

### Dried Plasma Spots

DPSs (30 μl) were punched out entirely with a 3-mm hand puncher and spiked with 0.23 ng of the deuterated internal standard [^2^H_4_]-5-CH_3_-H_4_folate. Subsequently, the mixture was suspended in 1 ml of extraction buffer 2 and placed in an ultrasonic bath with ice for 60 min in the dark. Afterward, the suspension was centrifuged at 4,000 rpm for 20 min at 4°C and subjected to SPE as described below.

### DBSs for Plasma 5-CH_3_-H_4_folate Determination (DBSPs)

DBSs (50 μl) were punched out entirely as described above with a 3-mm hand puncher, and the punched out discs were suspended in 1 ml of boiling extraction buffer 1 and immediately spiked with 0.39 ng of the internal standard [^2^H_4_]-5-CH_3_-H_4_folate. The suspension was heated in a water bath for 2 min at 100°C for enzyme denaturation. After cooling on ice, the samples were placed in an ultrasonic bath with ice for 60 min in the dark. Afterward, the samples were centrifuged at 4,000 rpm (20 min, 4°C), and the supernatant was subjected to SPE as described below.

### Solid-Phase Extraction

The DBS, DBSP, and DPS extracts were purified by SPE using a 12-port vacuum manifold (Merck) equipped with Strata SAX cartridges (quaternary amine, 100 mg, 1 ml). The cartridges were activated with two volumes of methanol and two volumes of equilibration buffer (pH 7), followed by the application of the respective DMS extracts. Afterward, the cartridges were washed with two volumes of equilibration buffer and subsequently dried by vacuum suction. 5-CH_3_-H_4_folate was eluted with 0.5 ml eluting solution.

## Optimization and Validation of the DBS Methodology

### Recovery of 5-CH_3_-H_4_folate from DBS after Extraction

To determine the step-by-step recoveries of 5-CH_3_-H_4_folate during the sampling and extraction processes, 12 DMSs were produced by adding absolute 11.8 ng 5-CH_3_-H_4_folate (514 nmol/L spot) by pipetting 30 μL of an 857 nmol/L aqueous solution on the pre-imprinted circles along with 50 μl of whole blood surrogate (see below). After 2 h of drying, three spots were punched out entirely with the 3-mm hand puncher to investigate the degree of elution during (1) 60 min of ultrasonication. The remaining nine DMS were used to assess the relative recoveries after (2) incubation at 37°C in a water bath under constant agitation for enzymatic digestion of the polyglutamates, (3) heating and centrifugation, and (4) SPE clean-up. To investigate the analyte loss rates, [^2^H_4_]-5-CH_3_-H_4_folate was added after each extraction step before analysis.

### Stability of DBSs

For this experiment, 12 DBSs were produced and stored with a desiccant at –20°C in resealable bags until analysis. Three spots were extracted on four different days in triplicate over 2 weeks.

### Recovery of H_4_folate in DBSs

For the H_4_folate analysis, 12 DBSs were produced and spiked with 145, 72.5, 48.3, 29, 14.5, 7.25, 4.8, and 2.9 pmol [^2^H_4_]-H_4_folate before extraction. After processing the samples according to the DBS extraction proceduredescribed above, all samples were analyzed for the labeled tetrahydrofolic acid to determine the matrix influence on the recovery. Venous blood samples (100, 500, 1,000, and 2,000 μl) and a DBS of a TT genotype were examined as described above with minor modifications. [^2^H_4_]-H_4_folate (1.33 ng) was added to DBS along with venous blood (100 μl). The remaining samples were spiked with double the standard amount.

### Comparison of DMSs with Conventional Blood and Plasma Samples

As there is no reference material available, we checked the validity of our methods by comparing DBS, DBSP, and DPS from finger blood and vP with conventional venous blood and vP samples. Aliquots of vP (50 μl), venous DPSs (30 μl), venous blood (50 μl), venous DBSs (50 μl), and DBSs from finger blood (50 μl) were extracted in triplicate for plasma 5- CH_3_-H_4_folate and whole blood 5-CH_3_-H_4_folate. The hematocrit value of the blood donor was determined to convert the results obtained for the plasma folate from DBSs. The plasma analyte concentration in DBSPs was determined as follows: *c*(*A*)_DBSP_/(1 –hematocrit/100%).

### Liquid Chromatography–Tandem Mass Spectrometry

Samples were measured by means of LC–MS/MS (Finnigan Surveyor Plus HPLC System and Triple Quadrupole TSQ Quantum Discovery Mass Spectrometer; Thermo Electron Corporation, Waltham, MA, USA). Analytes were separated on a Nucleosil 100–5 C18 EC 250/3 column (Macherey-Nagel, Düren, Germany).

Aqueous formic acid (0.1%; eluent A) and acetonitrile containing 0.1% formic acid (eluent B) were used as eluents at a flow rate of 0.3 ml/min. Gradient elution started at 0% B, followed by a linear increase of B to 10% within 2 min and to 20% within a further 15 min. Subsequently, the mobile phase was programmed to 100% B within a further minute and was held at 100% B for an additional 5 min before equilibrating the column for 14 min with the initial mixture. The multiple-reaction monitoring transitions of 5-CH_3_-H_4_folate are shown in Table 1.

**Table 1:** Multiple-reaction monitoring transitions of 5-CH_3_-H_4_folate.

Compound	Precursor ion (*m/z*)	Collison energy (V)	Product ion (*m*/*z*)
5-CH_3_-H_4_folate	460	21.0	313
	460	38.0	180
[^2^H_4_]-5-CH_3_-H_4_folate	464	21.0	317
	464	38.0	180

The first 12 min were diverted to waste. The spectrometer was operated in the positive electrospray mode using multiple-reaction monitoring (MRM). The spray voltage was set to 3,900 V, capillary temperature to 320°C, and the capillary voltage to 35 V.

### Limits of Detection and Quantification

LODs and LOQs for folates were determined according to a previous report [24]. For the LOD, LOQ, and recovery determination, blank matrices from a previous study [9] were used, with minor modifications. The plasma surrogate for DPS was produced as aforementioned. The blood surrogate consisted of a mixture of 100 ml plasma surrogate with an additional 15 g of lyophilized egg white, which represented the cellular fraction of blood. The spotting procedure followed the instructions detailed above. LC–MS/MS analysis of the blank surrogates confirmed the absence of the folate analyzed using this method. For LOD and LOQ determination, the matrices were spiked with 5-CH_3_-H_4_folate at four different concentrations, starting at slightly above the LOD and ranging 1–10-fold the amount of the analyte. After addition of the internal standard [^2^H_4_]-5-CH_3_-H_4_folate, all samples were processed as aforementioned and then analyzed by LC–MS/MS.

### High-Performance Liquid Chromatography with Diode Array Detection

The purity of 5- CH_3_-H_4_folate and [^2^H_4_]-5-CH_3_-H_4_folate used for obtaining an appropriate response were determined by measuring the analyte using HPLC-DAD according to the method from a previous study [25], with minor modifications. Folic acid was used as the internal standard. The HPLC-DAD system consisted of a Merck Hitachi L-7100 pump, L-7200 autosampler, and L-7455 diode array detector (Merck). The folates were separated on a Nucleosil 100–5 C18 EC 250/3 column (Macherey-Nagel). Gradient elution was performed with 0.1% aqueous acetic acid (eluent A) and methanol (eluent B). The starting conditions were 10% B held for 7 min, followed by a linear increase to 50% within 14 min, and then to 100% within a further 2 min, which was held for an additional 1 min. Following a linear decrease to the initial mixture within 2 min, 10% B was held for 9 min. 5-CH_3_-H_4_folate was detected at *λ*_max_ = 290 nm, and the calibration standards were prepared by diluting the 5-CH_3_-H_4_folate in extraction buffer 2. Folic acid (23 nmol) was used as the internal standard and was mixed with the analyte at molar ratios ranging from 1:15 to 1:1 for calibration. The 5-CH_3_-H_4_folate stock solution was used for further dilution and calibration.

### Calibration and Quantitation

Calibration solutions were prepared by mixing the internal standard solution with the corresponding analyte solution. The molar ratios of internal standard (S) and analyte (A) [*n*(*S*)/*n*(*A*)] were 3:1–1:35 for DBS calibration and 8.5:1–1:13.5 for DBSP and DPS calibration. For calculation of the calibration functions, linear regression was used by combining the molar ratios with the peak area ratios [*A*(*S*)/*A*(*A*)] from the LC–MS/MS measurements. The consistency of the responses was checked by measuring a randomly chosen *n*(*S*)/*n*(*A*) value in the linear range of the response functions.

### Precision

Inter-assay precision was determined by analyzing samples of DPSs and DBSs in triplicate, with three independent experiments over 2 weeks, while intra-assay precision was determined by injecting one sample three times in a row.

### Recoveries of SIDAs

Blank matrices of 50 μl DBSs (as described in theLODs and LOQs chapter) were spiked with 5.0, 14.4, and 28.8 pmol of 5-CH_3_-H_4_folate for whole blood 5-CH_3_-H_4_folate and with 0.7, 1.8, and 3.5 pmol of 5-CH_3_-H_4_folate for plasma 5-CH_3_-H_4_folate. Blank matrices of 30 μl dried plasma spots were spiked with 0.3, 0.7, and 1.4 pmol 5-CH_3_-H_4_folate. All samples were analyzed by SIDA as described above. The recovery was calculated as the mean of the addition experiments.

## Preliminary Applications in Human Studies

### 5-CH_3_-H_4_folate Bioavailability

To assess the sensitivity of the DPS method, a short-term bioavailability trial was conducted.

To saturate the body folate stores, a daily morning dose of 800 μg 5-CH_3_-H_4_folate (in capsule form) was given to one healthy male volunteer (27 years old, CT genotype) for 2 weeks. No further folate was administered 2 days before blood sampling. An additional overnight fasting plasma sample was obtained to determine the baseline plasma 5-CH_3_-H_4_folate level. After the administration of a further 400 μg of 5-CH_3_-H_4_folate, plasma samples were obtained after 10 defined timeframes. For the first 2 h, plasma samples were collected at increments of 20 min. After a further 80 min, the plasma was collected hourly for 2 h. The last plasma sample was obtained after a further 90 min. vP (30 μl) was immediately spotted onto preimpregnated filter cards as described above, and a further 60 μl of plasma was frozen in centrifuge tubes without additives until extraction.

### 5-CH_3_-H_4_folate Status

Eight volunteers (six females and two males) were recruited for DBS screening. The health status and lifestyle of the volunteers were not taken into account. Each subject delivered three DBSs, undergoing the procedure described above.

### Data Analysis

All data analysis was performed using Xcalibur Software from Thermo Scientific (Waltham, MA, USA).

## Results and Discussion

SIDAs proved to be a good choice for the accurate and precise quantitation of folates, as the steady loss of vitamins in the samples is compensated for by the addition of the stable isotope-labeled standards. A promising alternative to conventional venous blood sampling is DBS sampling, as no medical surveillance is necessary, storage costs can be reduced, and DBS devices can be easily shipped. Therefore, these methods may be particularly valuable for human studies with many subjects. Nevertheless, the small sample amounts and complexity of the matrix are challenging in the minor blood component analysis. Therefore, we decided to combine this type of sampling with the advantages of an SIDA to reduce the required sample volume from 400 μl to 30 μl of plasma, as compared to our standard procedure [9], and to 50 μl of whole blood for each sample.

### Results of Calibration for SIDAs

The calibration curves for 5-CH_3_-H_4_folate were linear in terms of the applicable molar ratios, which ranged from 1:33.3 to 1.5:1 (*y* = 0.8991*x*−0.0055, *R* ^2^ = 0.9999) for whole blood analysis in DBS and from 1:6.7 to 3.4:1 (*y* = 0.7995*x*−0.0219, *R* ^2^ = 0.9994) for plasma analysis in DBS. The response function for DPS is represented by a polynominal function (*x* ^3^), with a molar ratio ranging from 1:10 to 3.4:1 (*y* = 0.0192*x* ^3^–0.1842*x* ^2^ + 1.6096*x*−0.0143, *R* ^2^ = 0.9997). The response equations for the linear functions were calculated as follows:

$$A(\text{labeled standard})/A(\text{analyte})=R_{\text{F}}\;\times \;n(\text{labeled standard})/n(\text{analyte})+b$$

For the polynominal function (labeled standard: IST, analyte: *A*) we used the following equation:

$$n\left(\text{IST}\right)/n\left(A\right)\;=m_{1}\times A\left(\text{IST}\right)/A{\left(A\right)}^{3}+m_{2}\times A\left(\text{IST}\right)/A{\left(A\right)}^{2}+m_{3}\times A\left(\text{IST}\right)/A\left(A\right)\;+b$$

Optimization and validation of the DMS methodology

### Results of Recovery Studies for 5-CH3-H4folate from DBS after Extraction

**Fig 1:**
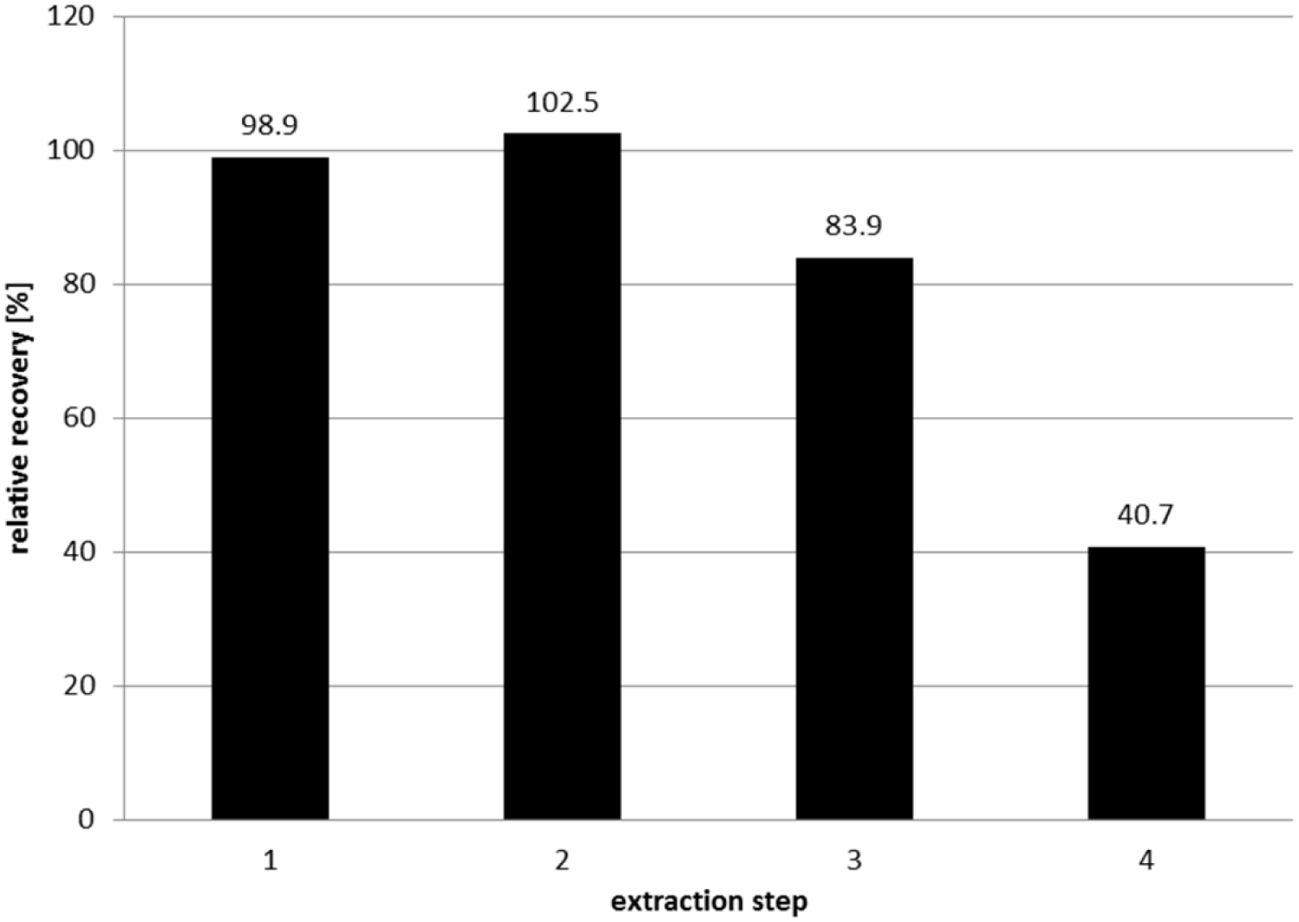
Step-by-step recovery of 5-CH_3_-H_4_folate during extraction after (1) 60-min ultrasonication treatment, (2) incubation at 37°C in a water bath under constant agitation for enzymatic digestion of the polyglutamates, (3) heating, and (4) solid-phase extraction clean-up.

The results of the step-by-step recoveries are shown in Fig 1.

The relative recoveries were 98.9%, 102.5%, 83.9%, and 40.7% after (i) 60 min of ultrasonication treatment, (ii) incubation at 37°C in a water bath under constant agitation for enzymatic digestion of the polyglutamates, (iii) heating, and (iv) SPE clean-up, respectively. Drying at room temperature allows the matrix to enclose the analyte molecules without lysis of the erythrocytes and to protect them against oxidation by air. The ultrasonication treatment was sufficient for resolving nearly all the analytes added. The method for quantifying the whole blood folate requires enzymatic digestion of the pteroyl polyglutamates. Maintenance of the activity of the enzymes added for digestion along with sufficient cell lysis of the erythrocytes requires the use of a nondenaturating tenside. Both requirements were met by adding Triton X-100 to the extraction buffer, the efficiency of which was clearly observed during extraction by the color change from red to white on the cellulose filter card. After the incubation step, no polyglutamates were detected, thus confirming the enzymatic activity during the deconjugation step. The initial losses of 5-CH_3_-H_4_folate (16.1%) are due to the heating step after the incubation procedure, and a further loss of 43.2% can be ascribed to the SPE clean-up. However, these losses are compensated for by the addition of the labeled standards.

### Stability of Folates in DBSs

**Fig 2:**
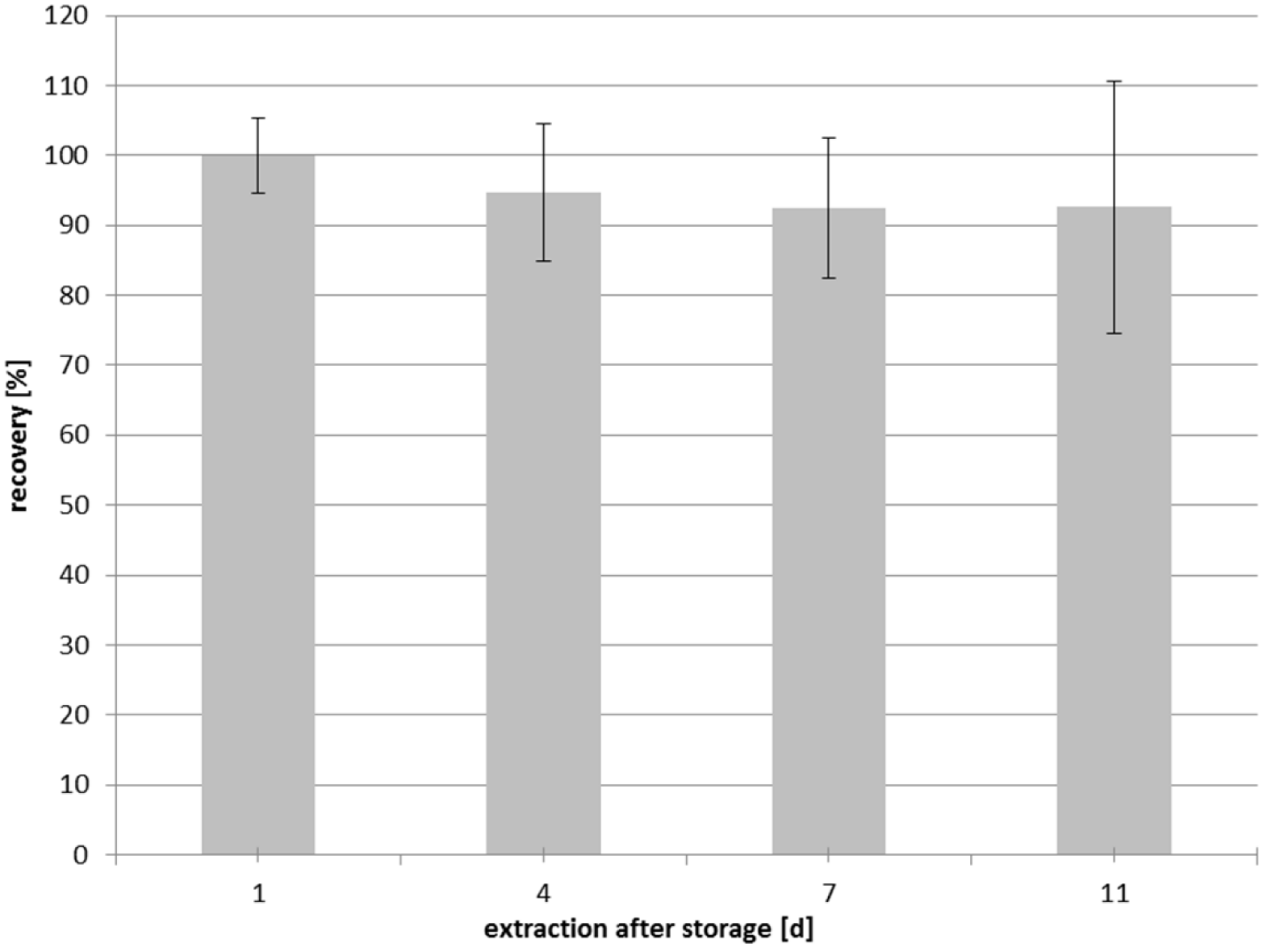
Stability of dried blood spots during storage (–20°C).

Folate stability testing during storage at –20°C revealed no significant decrease in 5-CH_3_-H_4_folate concentration (Fig 2) over a period of 11 days (*P* > 0.05, two-sided *t*-test).

These observations are consistent with the findings from a study conducted by O’Broin and Gunter [20], who determined 93.5% ± 4.2% recovery in a finger prick DBS folate test after 12 months’ storage at –20°C.

### Recovery of H_4_folate from DBSs

**Fig 3:**
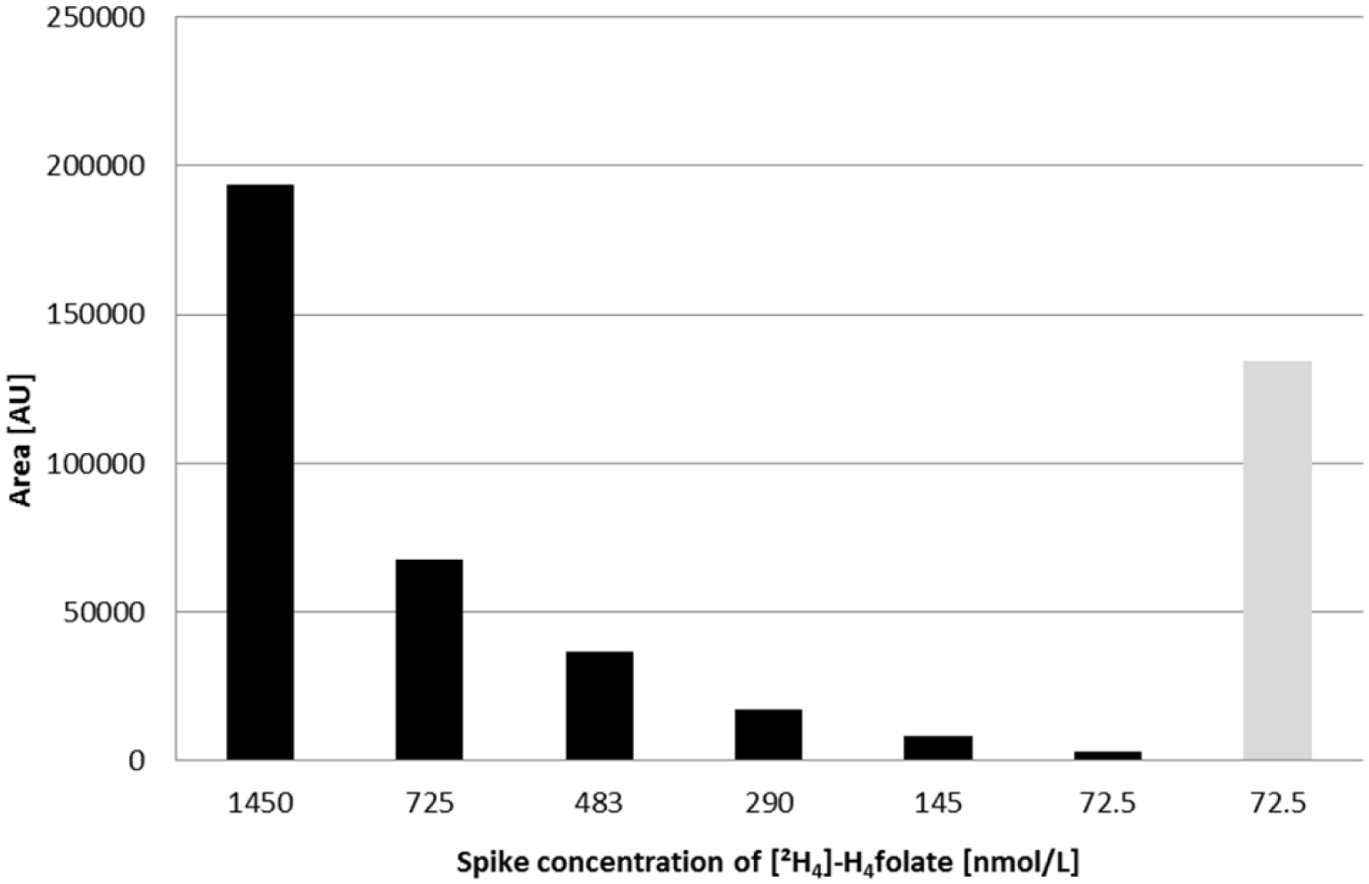
Recovered areas of different spiked concentrations of [^2^H_4_]-H_4_folate in dried blood spots after extraction (black bars) compared with a reference solution (gray bar).

Unfortunately, during preliminary screening with the optimized extraction procedures, H_4_folate was not detected in DBSs (data not shown). No H_4_folate was detected even in the venous blood samples and DBSs of a volunteer who was homozygous for the MTHFR polymorphism, although a decisive part of the volunteer’s folate level should be present as this vitamer. To clarify this observation, we spiked DBSs with different amounts of [^2^H_4_]-H_4_folate and found a substantial decrease in the concentration of this folate during extraction (Fig 3).

The respective signals at the two lower spiking levels, that is, 72.5 and 145 nmol/L, were barely distinguishable from the background noise. Compared with the signal for a 72.5 nmol/L solution of [^2^H_4_]-H_4_-folate (gray bar in Fig 3), the area of the signal after extraction from the dried blood decreased by a factor of 42. Since prior degradation during the drying process was not taken into account, even higher losses should be expected. These findings may be due to the impact of oxygen on H_4_folate [10]. Folates may be particularly sensitive during this procedure due to their reactivity with blood glucose upon heating [26]. Thus, a decrease in folate and increase in degradation products, particularly carboxyethyl derivatives, due to the application of heat can be assumed.

As most of the estimated concentrations of H_4_folate in erythrocytes, especially for the CC and CT genotypes of the MTHFR polymorphism, are below 290 nmol/L [13,27], predictions of individual genotypes based on 5-CH_3_-H_4_folate/H_4_folate ratios are difficult as H_4_folate levels below 290 nmol/L H_4_folate hardly were detectable in DBS.

### 5-CH_3_-H_4_folate Analysis in DBSs for Whole Blood Folate

Folates are ubiquitous in human blood and plasma samples, and red blood cells are especially rich in folates. However, to assess LOD and LOQ, matrices free from these analytes are required, and therefore, a surrogate matrix to blood needed to be developed. Thus, in accordance with the method by Mönch et al. [9], a whole blood surrogate was designed with lyophilized egg white representing erythrocytes (equivalent to 90% hemoglobin) and cellular components mixed with water, sunflower oil, and sodium chloride as the plasma surrogate. According to a previous study [28], the cellular fraction (egg white) was calculated with a hematocrit of 40%, corresponding to 15 g of lyophilized egg white in the 100 ml plasma surrogate.

Using the spiking procedure proposed by Hädrich and Vogelgesang [24], the LOD and LOQ for 5-CH_3_-H_4_folate were 9.1 and 27 nmol/L, respectively (Table 2).

**Table 2:** Validation Data of stable isotope dilution assays.

Sample	Intra-assay precision ^a^	Inter-assayprecision ^a^	Recovery ^b^ (%)	LOD	LOQ
	CV (%)	CV (%)		(nmol/L)	(nmol/L)
DBS	8 (287 ± 24.1 nmol/L)	3 (263 ± 6.8 nmol/L)		9.1	27
(Level 1)			94 (576 nmol/L)		
(Level 2)			109 (288 nmol/L)		
(Level 3)			106 (99 nmol/L)		
DBSP	16 (20.6 ± 2.7 nmol/L)	13 (22.2 ± 3.5 nmol/L)		2.2	6.3
(Level 1)			111 (69 nmol/L)		
(Level 2)			116 (35 nmol/L)		
(Level 3)			110 (14 nmol/L)		
DPS	9 (17.4 ± 1.5 nmol/L)	4 (21.0 ± 0.9 nmol/L)		1.5	4.4
(Level 1)			87 (46 nmol/L)		
(Level 2)			92 (23 nmol/L)		
(Level 3)			80 (10 nmol/L)		

DBS, dried blood spot; DBSP, dried blood spots for plasma folate; DPS, dried plasma spot; LOD, limit of detection; LOQ, limit of quantification.

^a^ values in brackets: plasma and blood 5-CH_3_-H_4_folate levels from one volunteer

^b^ values in brackets: spiking levels for plasma and blood 5-CH_3_-H_4_folate recoveries

**Fig 4:**
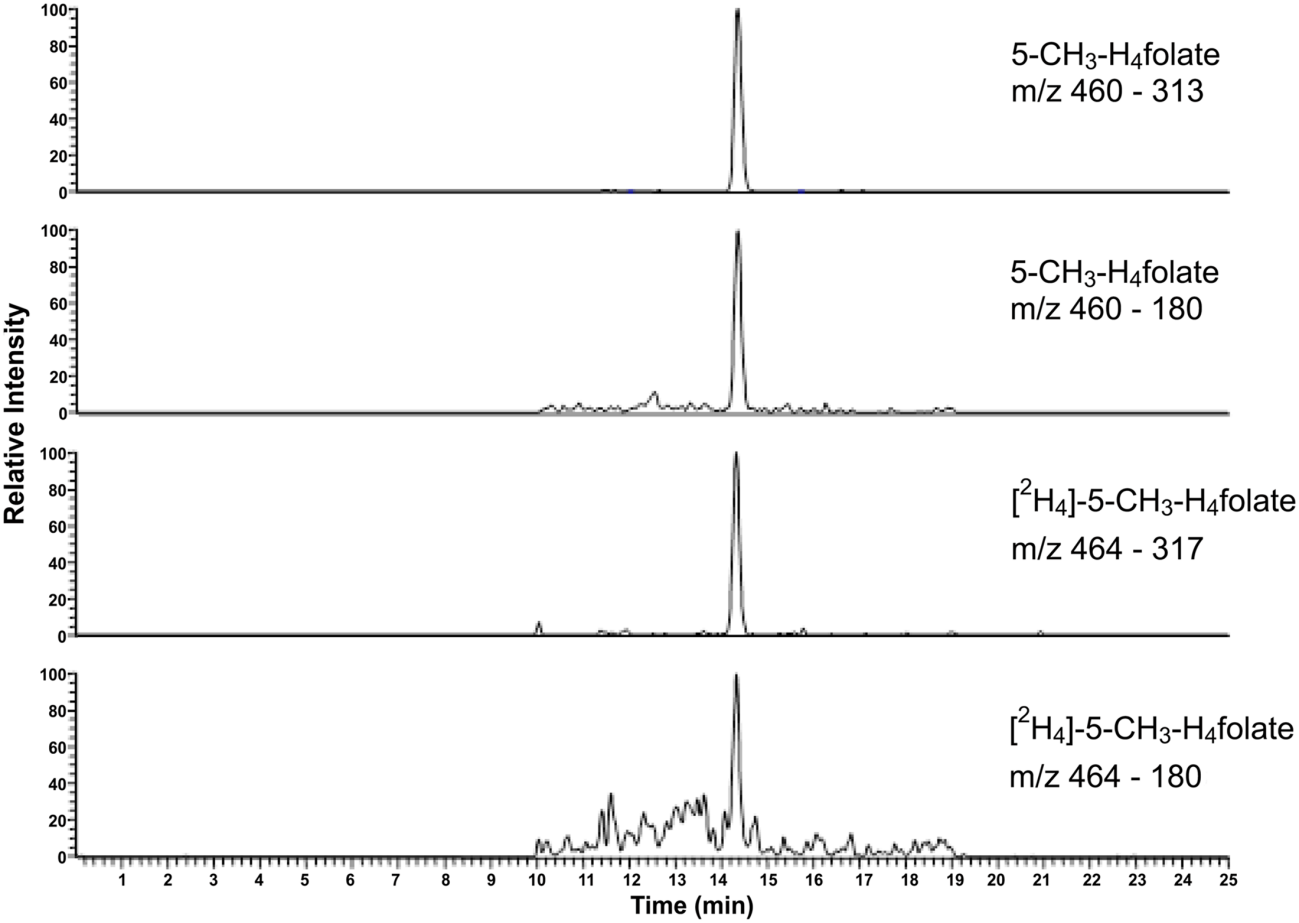
Liquid chromatography–tandem mass spectrometry chromatogram in the multiple-reaction monitoring mode for the dried blood spots.

The recovery of 5-CH_3_-H_4_folate for the complete SIDA was determined by spiking the blank surrogate with specific amounts of this analyte and performing SIDA with SPE clean-up, as detailed above. The recoveries were 94%, 109%, and 106% for the three spiking levels shown in Table 2. Thus, the LOD was slightly lower than for the method published previously [9]. However, our method would allow material costs to be minimized due to the smaller amount of chemicals used compared with that in the commonly applied method of erythrocyte extraction. The length of time required for the incubation step was reduced from 16 to 4 h, as the pteroyl polyglutamates of the 5-CH_3_-H_4_folate could no longer be detected in the sample extracts after incubation (data not shown). The small amount of 0.5 ml elution buffer further ensured the preservation of the sensitivity of this method. A typical MRM chromatogram for DBS analysis is shown in Fig 4.

### Folates Analysis in the Dried Plasma Spots for Plasma Folate

Blood sampling usually gives 5–10 ml of venous blood. In a method reported previously, 400 μl of plasma was used for folate determination [9]. In contrast, the method presented here reduced this volume to 30 μl for the quantitation of the main vitamer 5-CH_3_-H_4_folate in plasma. Preimpregnation of the absorber material with 1% aqueous ascorbic acid helped to stabilize the folate as reported by O’Broin and Gunter [22]. As plasma does not contain any cells, we did not add tenside Triton X as an elution agent during ultrasonication. The following steps are similar to the extraction procedure published by Mönch et al. [9], which revealed sufficient sensitivity. As the sample amount was reduced by a factor of 13, downsizing the subsequent buffer volumes and SPE cartridge size also resulted in a faster clean-up procedure.

To assess the recoveries and LOD/LOQ, we used a surrogate containing specific amounts of all the main components (protein, fat, sodium chloride, and water) in the plasma samples. In contrast to the widespread use of diluted plasma in clinical research, our approach mimics the matrix effects more realistically. Finally, the LOD and LOQ were 1.5 and 4.4 nmol/L 5-CH_3_-H_4_folate, respectively.

The recoveries for 5- CH_3_-H_4_-folate were 87%, 92%, and 80% for the three different spiking concentrations detailed in Table 2.

### Folates Analysis in DBSs for Plasma Folate

Plasma folate analysis in DBSs is challenging due to the presence of plasma conjugase. The enzyme remains active after adding reducing agents and buffer solution to DBSs [20]. Thus, the intracellular pteroyl polyglutamates are deconjugated to their respective monoglutamates after cellular degradation, which eventually leads to an artifactual increase of the measured plasma folate level. This interference was avoided by introducing a heating step with boiling extraction buffer. Thus, all the enzymes were inactivated instantaneously, and polyglutamate deconjugation was prevented.

Using the same whole blood matrix surrogate detailed above, the LOD and LOQ for 5-CH_3_-H_4_folate were 2.2 and 6.3 nmol/L, respectively. These concentrations were slightly higher than those found in the DPS experiment, which could be attributed to the increased interference of the additional protein with the analyte. Compared with DBSs, the LOD of DPSs was lower because the deconjugation step was omitted, and thus, generation of the interfering low-molecular-weight degradation products was mostly prevented.

The recoveries for 5-CH_3_-H_4_folate in this SIDA were 111%, 116%, and 110% for three different spiking concentrations detailed in Table 2. These elevated recovery levels could potentially be attributed to the higher viscosity of the whole blood surrogate, which formed a glassy film on the card. This film is caused by the egg white component and may help to protect and stabilize the analyte molecules. As a result, the deuterated standard added to the boiling suspension may have degraded slightly more than the analyte molecules trapped in the protein matrix, thus resulting in recoveries higher than 100%.

### Precision of the New Assays for 5-CH_3_-H_4_folate

Intra-assay precision was assessed by multiple injection (*n* = 3) of identical extracts prepared on the same day. Further, inter-assay precision was determined by extraction of samples on three different days over 2 weeks, with each analysis performed in triplicate (Table 2). For the multi-injection assay, CVs in DBSs, DBSPs, and DPSs were 8%, 16%, and 9% for 5-CH_3_-H_4_folate, respectively, while the CVs of the inter-assay studies were 3%, 13%, and 4%, respectively.

### Comparison of DMSs with Conventional Blood and Plasma Sampling

**Fig 5:**
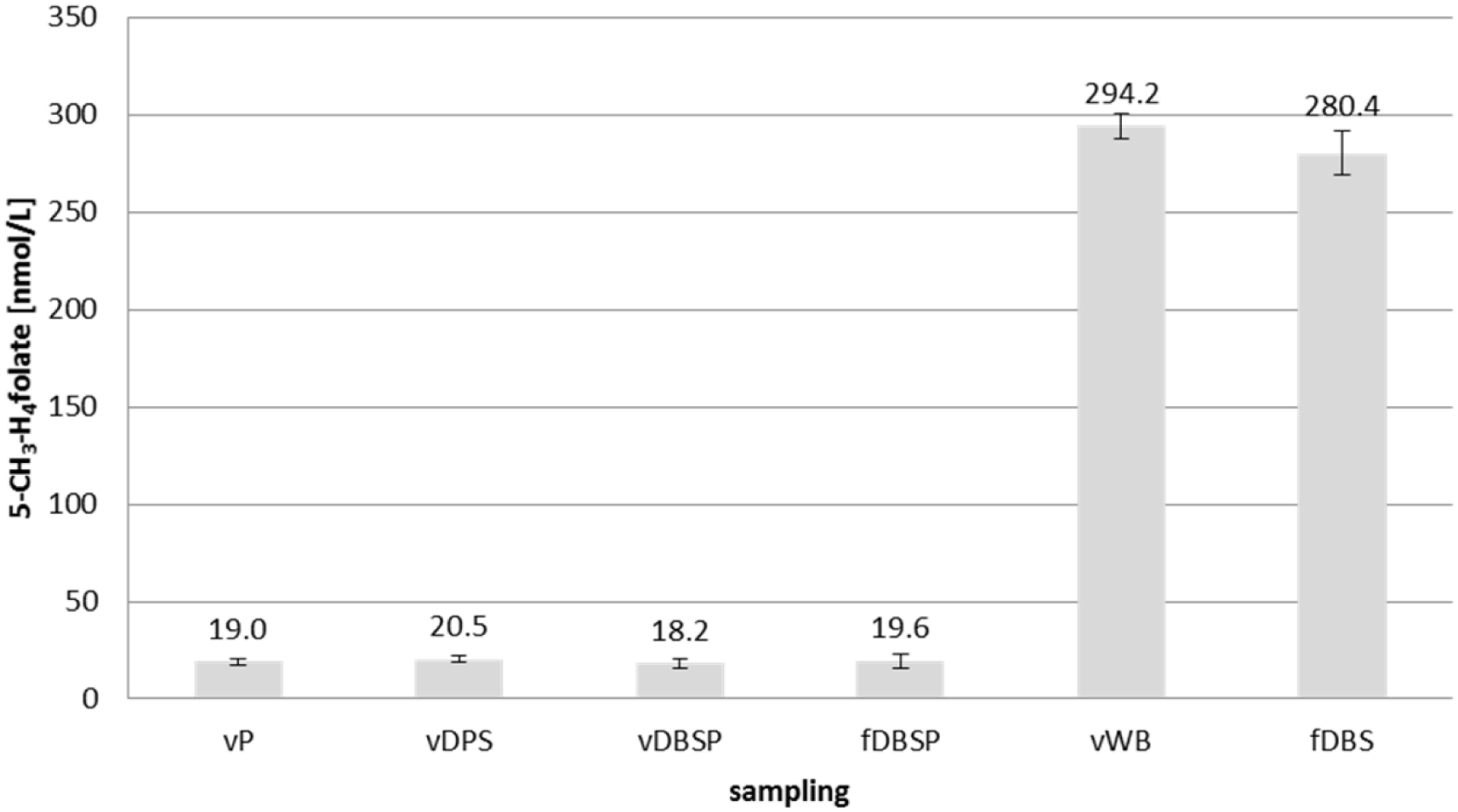
Comparison of the developed dried matrix spot methods with conventional sampling procedures. vP plasma sample from venous blood, vDPS dried plasma spot from venous blood, vDBSP dried blood spot with plasma folate determination from venous blood, fDBSP dried blood spot with plasma folate determination from finger blood, vWB whole blood sample from venous blood, fDBS dried blood spot from finger blood.

Three different sampling methods were compared with the newly developed DMS methods, and the results are depicted in Fig 5.

Plasma 5-CH_3_-H_4_folate levels of DPSs and DBSs were compared after conversion of 5-CH_3_-H_4_folate from DBSs to plasma folate using the hematocrit value of the blood donor (45%) and the formula shown above. The results from traditional vP sampling showed no significant differences (two-sided *t*-test) compared with DPS or DBSP sampling from venous (v) or finger blood (f). As all of these four sampling procedures led to the same results, the accuracy of the DPS and DBSP methods was confirmed. There were no significant differences in plasma folate in the DBSP between venous and finger blood (*P* > 0.05). Venous whole blood and DBSs from finger blood exhibited very similar levels of 5-CH_3_-H_4_folate to that in whole blood. The percentage deviation between the two values was 4.7%, which was not statistically significant (*P* > 0.05); thus, no difference was found between venous and capillary blood. For these methods, the whole blood spot is used for 5-CH_3_-H_4_folate status determination to prevent uneven folate distributions between the center and peripheral parts of the DMS. These inhomogeneities may be due to folate–protein interactions or higher levels of erythrocytes in the center of the spot as a result of chromatographic effects during the spreading of the blood drop on the filter card.

## Preliminary Applications in Human Studies

### Bioavailability of 5-CH_3_-H_4_folate

**Fig 6:**
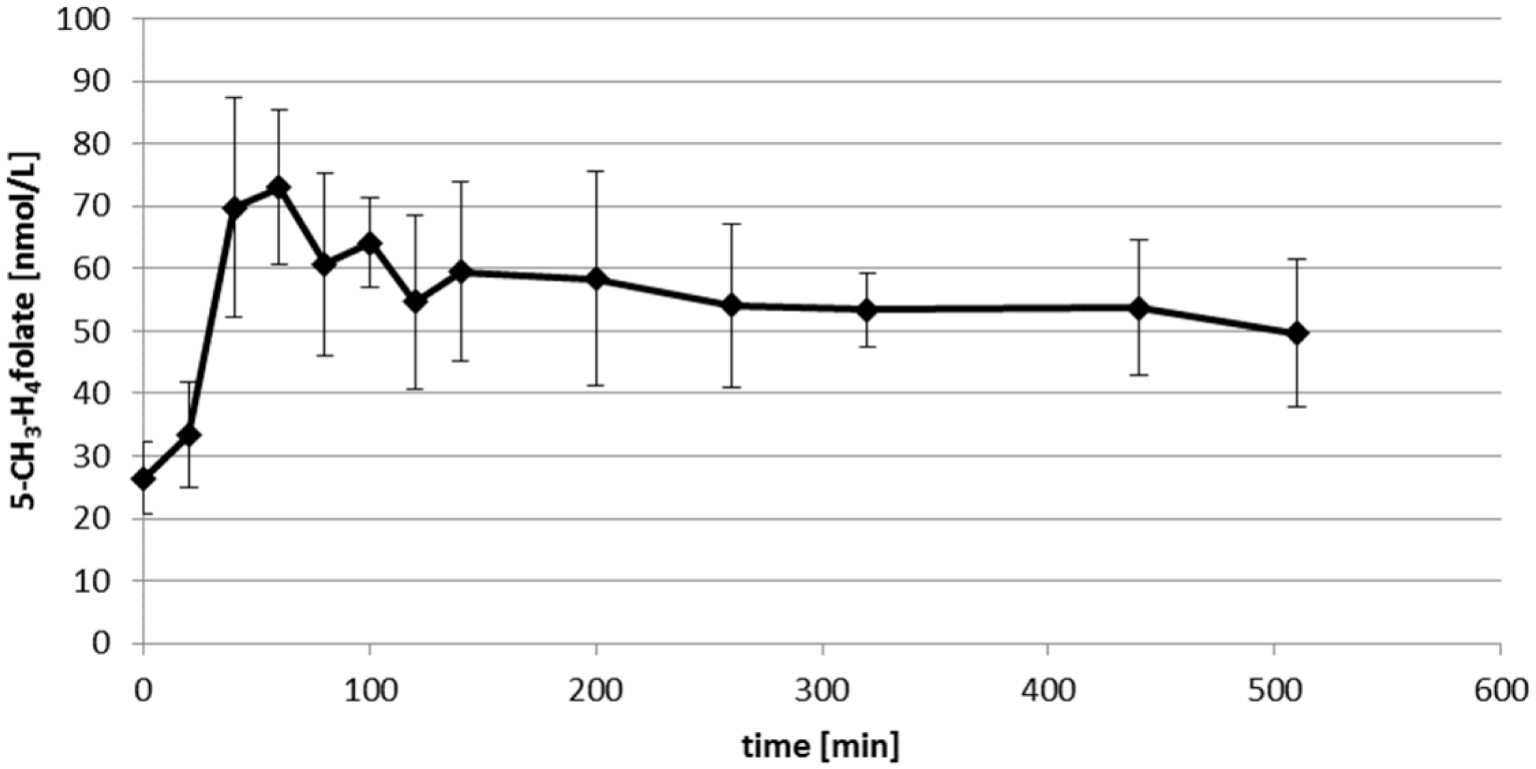
Plasma level of 5-CH_3_-H_4_folate in 30 μl dried plasma spots after uptake of 400 μg 5-CH_3_-H_4_folate.

**Fig 7:**
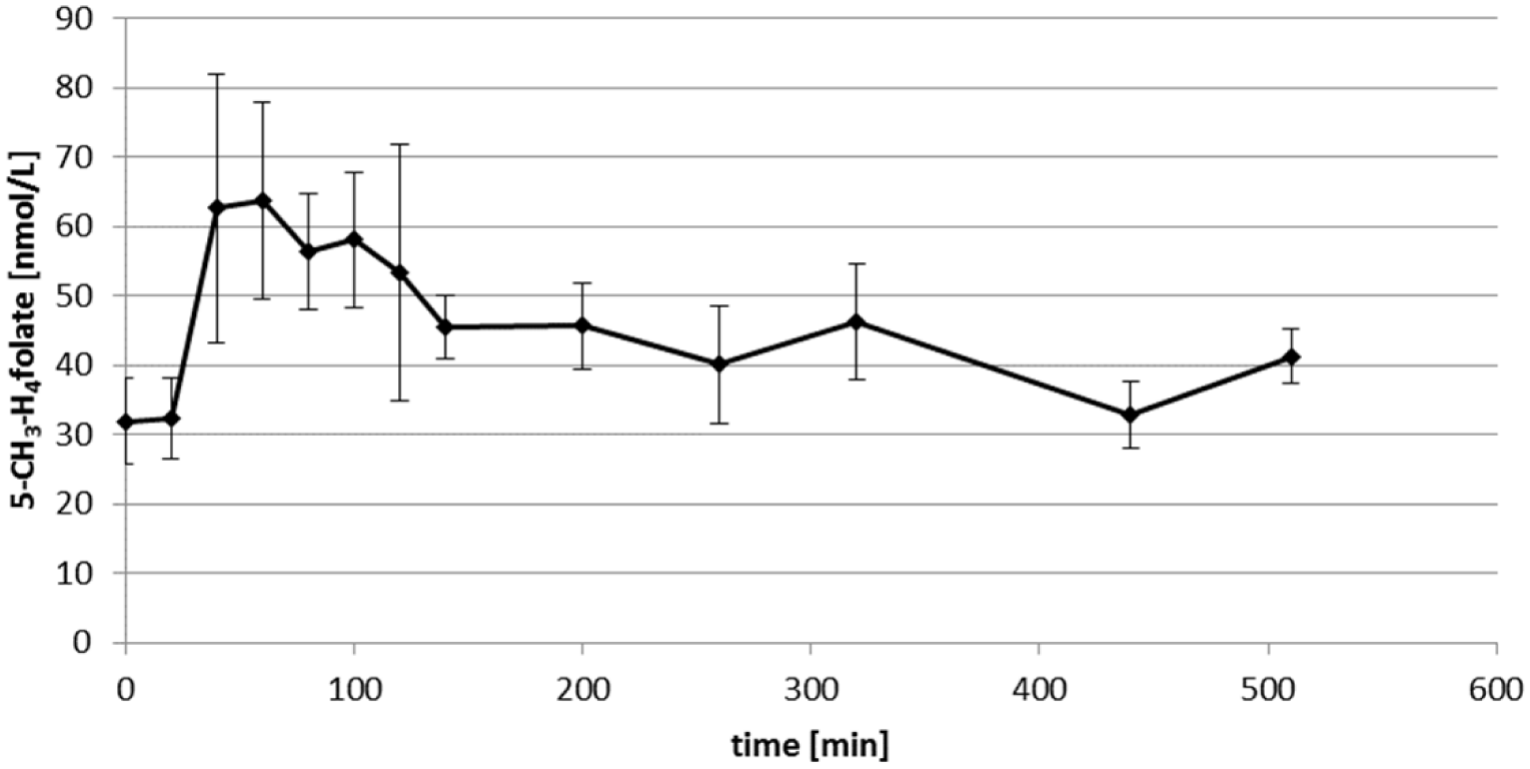
Plasma level of 5-CH_3_-H_4_folate in 60 μl of plasma at different sampling times after uptake of 400 μg 5-CH_3_-H_4_folate.

Bioavailability studies with DPS are advantageous over conventional plasma analysis due to the low sample volumes and sufficient stability [22]. In our pilot study, venous blood samples were taken after one volunteer took a single oral dose of 400 μg 5-CH_3_-H_4_folate. From these samples, DPS and conventional plasma was analysed. Figs 6 and 7 shows the curves from the DPS and plasma results.

Within the first 60 min, DPS folate had increased from a baseline level of 26 ± 6 to 72 ± 12 nmol/L, followed by a steady decrease to 50 ± 12 nmol/L after 510 min. No significant difference was found compared with the plasma curve (*P* > 0.05). Due to the lower accuracy of volume adjustment in capillary sampling, a minimum of three DPS or 60 μl aliquots of plasma is needed for analysis. However, sensitive bioavailable 5-CH_3_-H_4_folate determination is possible despite these low sample volumes. As the required amount of sample is reduced for folate quantification, the remaining volume of the sample could be used for quantifying additional metabolites of clinical relevance such as homocysteine.

### 5-CH_3_-H_4_folate Status

**Fig 8:**
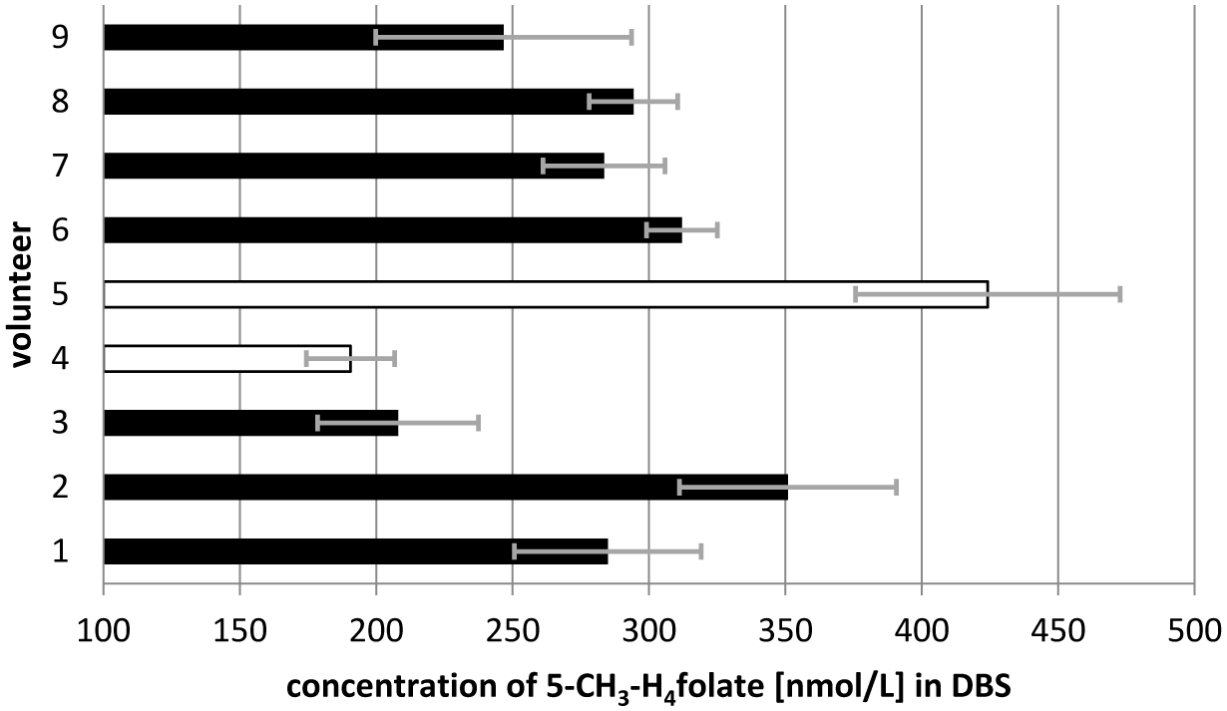
Screening of whole blood 5-CH_3_-H_4_folate in whole blood dried blood spots from eight volunteers.

The recommended daily intake (RDI) of folate depends on age and lifestyle. For adults in Germany, the RDI is 300 μg/d of dietary folate equivalents (DFE) for males and females, 550 μg/d DFE for women during pregnancy, and 450 μg/g DFE during lactation [29,30]. The mean dietary intake in countries without mandatory fortification such as Germany is quite lower than these daily recommendations. Eight volunteers were screened to assess the folate status in a small cohort of Germans, and the inter-individual variety of 5-CH_3_-H_4_folate blood concentrations is shown in Fig 8.

The black bars depict the levels of the eight randomly chosen female and male participants (age range 26–30 years). In contrast, the white bars show the whole blood levels from a volunteer homozygous for the MTHFR polymorphism before (4) and after (5) supplementation with folic acid for 8 weeks. Compared with the mean threshold for sufficient erythrocyte folate (≥340 nmol/L [29], ≥326 nmol/L 5-CH_3_-H_4_folate), all whole blood 5-CH_3_-H_4_folate levels exceed this value when converted to erythrocyte folate.

## Conclusions

The SIDAs presented in this research allow the reduction of sample volumes and the facilitation of extensive screenings for 5-CH_3_-H_4_folate in whole blood and plasma. The LODs and LOQs are sufficient for blood and plasma level determination in subjects with different MTHFR genotypes. However, more efforts are necessary to enable quantitation of H_4_folate. The 5-CH_3_-H_4_folate/H_4_folate ratio might contribute to the individual MTHFR genotype determination, and more research is warranted in this area. A solid and effective method for hematocrit assessment in DBSs needs to be established to enable clinical analysts to convert whole blood folate from DBSs to erythrocyte folate and vice versa.

## Supporting Information

S1 Table Data of Fig 2. Stability of dried blood spots during storage (–20°C). (DOCX)

S2 Table Data of Fig 3. Recovered areas of different spiked concentrations of [^2^H_4_]-H_4_folate in dried blood spots after extraction (black bars) compared with a reference solution (gray bar). (DOCX)

S3 Table Data of Fig 5. Comparison of the developed dried matrix spot methods with conventional sampling procedures. vP plasma sample from venous blood, vDPS dried plasma spot from venous blood, vDBSP dried blood spot with plasma folate determination from venous blood, fDBSP dried blood spot with plasma folate determination from finger blood, vWB whole blood sample from venous blood, fDBS dried blood spot from finger blood.
(DOCX)

S4 Table Data of Fig 6. Plasma level of 5-CH_3_-H_4_folate in 30 μl dried plasma spots after uptake of 400 μg 5-CH_3_-H_4_folate. (DOCX)

S5 Table Data of Fig 7. Plasma level of 5-CH_3_-H_4_folate in 60 μl of plasma at different sampling times after uptake of 400 μg 5-CH_3_-H_4_folate. (DOCX)

S6 Table Data of Fig 8. Screening of whole blood 5-CH_3_-H_4_folate in whole blood dried blood spots from eight volunteers. (DOCX)
