# Supplementary material for: Quantitation of 5-Methyltetrahydrofolic Acid in Dried Blood Spots and Dried Plasma Spots by Stable Isotope Dilution Assays
Source: PLoS One. 2015 Nov 25;10(11):e0143639. doi: 10.1371/journal.pone.0143639 (PMC4659665; doi:10.1371/journal.pone.0143639)
Supplement: S1 Table — (DOCX) [file pone.0143639.s001.docx]

Supporting Information

**S1-Table. (Data of Fig. 2. Stability of dried blood spots during storage (–20°C).)**

| day | Recovery [%] | ± SD [%] |
| --- | --- | --- |
| 1 | 100 | 5.3 |
| 4 | 94.7 | 9.9 |
| 7 | 92.5 | 10.0 |
| 11 | 92.9 | 17.5 |
