# Supplementary material for: Quantitation of 5-Methyltetrahydrofolic Acid in Dried Blood Spots and Dried Plasma Spots by Stable Isotope Dilution Assays
Source: PLoS One. 2015 Nov 25;10(11):e0143639. doi: 10.1371/journal.pone.0143639 (PMC4659665; doi:10.1371/journal.pone.0143639)
Supplement: S2 Table — (DOCX) [file pone.0143639.s002.docx]

Supporting Information

**S2-Table. (Data of Fig. 3. Recovered areas of different spiked concentrations of [^2^H_4_]-H_4_folate in dried blood spots after extraction (black bars) compared with a reference solution (gray bar).)**

| Spike solution | Spike concentration  [^2^H_4_]-H_4_folate [nmol/L] | Area [AU] |
| --- | --- | --- |
| 1 | 1450 | 193739 |
| 2 | 725 | 67783 |
| 3 | 483 | 36414 |
| 4 | 290 | 17239 |
| 5 | 145 | 8346 |
| 6 | 72.5 | 3192 |
| reference | 72.5 | 134148 |
