# Supplementary material for: Quantitation of 5-Methyltetrahydrofolic Acid in Dried Blood Spots and Dried Plasma Spots by Stable Isotope Dilution Assays
Source: PLoS One. 2015 Nov 25;10(11):e0143639. doi: 10.1371/journal.pone.0143639 (PMC4659665; doi:10.1371/journal.pone.0143639)
Supplement: S3 Table — vP plasma sample from venous blood, vDPS dried plasma spot from venous blood, vDBSP dried blood spot with plasma folate determination from venous blood, fDBSP dried blood spot with plasma folate determination from finger blood, vWB whole blood sample from venous blood, fDBS dried blood spot from finger blood. (DOCX) [file pone.0143639.s003.docx]

Supporting Information

**S3-Table. (Data of Fig. 5.** **Comparison of the developed dried matrix spot methods with conventional sampling procedures.** vP plasma sample from venous blood, vDPS dried plasma spot from venous blood, vDBSP dried blood spot with plasma folate determination from venous blood, fDBSP dried blood spot with plasma folate determination from finger blood, vWB whole blood sample from venous blood, fDBS dried blood spot from finger blood)

| Sampling method | c(5-CH_3_-H_4_folate) [nmol/L] | ± SD [nmol/L] |
| --- | --- | --- |
| vP | 19.0 | 1.7 |
| vDPS | 20.5 | 1.8 |
| vDBSP | 18.2 | 3.8 |
| fDBSP | 19.6 | 6.5 |
| vWB | 294.2 | 25.3 |
| fDBS | 280.4 | 20.4 |
