# Supplementary material for: Quantitation of 5-Methyltetrahydrofolic Acid in Dried Blood Spots and Dried Plasma Spots by Stable Isotope Dilution Assays
Source: PLoS One. 2015 Nov 25;10(11):e0143639. doi: 10.1371/journal.pone.0143639 (PMC4659665; doi:10.1371/journal.pone.0143639)
Supplement: S4 Table — (DOCX) [file pone.0143639.s004.docx]

Supporting Information

**S4-Table. (Data of Fig. 6. Plasma level of 5-CH_3_-H_4_folate in 30 µl dried plasma spots after uptake of 400 µg 5-CH_3_-H_4_folate.)**

| time [min] | c(5-CH_3_-H_4_folate) in DPS [nmol/L] | ± SD [nmol/L] |
| --- | --- | --- |
| 0 | 26 | 6 |
| 20 | 33 | 8 |
| 40 | 70 | 18 |
| 60 | 72 | 12 |
| 80 | 61 | 15 |
| 100 | 64 | 7 |
| 120 | 55 | 14 |
| 140 | 60 | 14 |
| 200 | 58 | 17 |
| 260 | 54 | 13 |
| 320 | 53 | 6 |
| 440 | 54 | 14 |
| 510 | 50 | 12 |
