# Supplementary material for: Quantitation of 5-Methyltetrahydrofolic Acid in Dried Blood Spots and Dried Plasma Spots by Stable Isotope Dilution Assays
Source: PLoS One. 2015 Nov 25;10(11):e0143639. doi: 10.1371/journal.pone.0143639 (PMC4659665; doi:10.1371/journal.pone.0143639)
Supplement: S5 Table — (DOCX) [file pone.0143639.s005.docx]

Supporting Information

**S5-Table. (Data of Fig. 7. Plasma level of 5-CH_3_-H_4_folate in 60 µl of plasma at different sampling times after uptake of 400 µg 5-CH_3_-H_4_folate.)**

| time [min] | c(5-CH_3_-H_4_folate) Plasma [nmol/L] | ± SD [nmol/L] |
| --- | --- | --- |
| 0 | 32 | 6 |
| 20 | 32 | 6 |
| 40 | 63 | 19 |
| 60 | 64 | 14 |
| 80 | 56 | 8 |
| 100 | 58 | 10 |
| 120 | 53 | 18 |
| 140 | 45 | 4 |
| 200 | 46 | 6 |
| 260 | 40 | 8 |
| 320 | 46 | 8 |
| 440 | 33 | 5 |
| 510 | 41 | 4 |
