# Supplementary material for: Quantitation of 5-Methyltetrahydrofolic Acid in Dried Blood Spots and Dried Plasma Spots by Stable Isotope Dilution Assays
Source: PLoS One. 2015 Nov 25;10(11):e0143639. doi: 10.1371/journal.pone.0143639 (PMC4659665; doi:10.1371/journal.pone.0143639)
Supplement: S6 Table — (DOCX) [file pone.0143639.s006.docx]

Supporting Information

**S6-Table. (Data of Fig. 8. Screening of whole blood 5-CH_3_-H_4_folate in whole blood dried blood spots from eight volunteers.)**

| volunteer | c(5-CH_3_-H_4_folate) [nmol/L] | ± SD [nmol/L] | remarks |
| --- | --- | --- | --- |
| 1 | 284.9 | 31.3 |  |
| 2 | 350.9 | 36.3 |  |
| 3 | 208.0 | 26.9 |  |
| 4 | 190.5 | 14.8 | T/T genotype before supplementation with folic acid |
| 5 | 424.2 | 44.3 | T/T genotype after supplementation with folic acid |
| 6 | 312.0 | 11.8 |  |
| 7 | 283.5 | 19.8 |  |
| 8 | 294.3 | 14.8 |  |
| 9 | 246.7 | 42.8 |  |
